# Association Between Dietary Patterns, Work Stress, Physical Activity, and Cardiometabolic Markers Among University Workers in Ghana: A Cross‐Sectional Study

**DOI:** 10.1002/hsr2.72986

**Published:** 2026-08-05

**Authors:** Daniel Ayeltigah, Mary Amoako, Richard Oguah, Dorcas Kafui Ledi, Adwoa Gyamfi, Michael Akenteng Wiafe

**Affiliations:** ^1^ Department of Biochemistry and Biotechnology, Faculty of Biosciences, College of Science Kwame Nkrumah University of Science and Technology Kumasi Ghana; ^2^ School of Nursing and Midwifery, College of Health Sciences Kwame Nkrumah University of Science and Technology Kumasi Ghana; ^3^ Department of Dietetics, School of Allied Health Sciences University of Development Studies Tamale Ghana

**Keywords:** cardiometabolic markers, dietary patterns, physical activity, work stress

## Abstract

**Background and Aims:**

Emerging evidence highlight metabolic markers as key indicators of health status, linking subtle metabolic changes with the rising global rate of non‐communicable diseases. Therefore, this study aimed to investigate the relationship between lifestyle and metabolic markers among university workers in Ghana.

**Methods:**

A cross‐sectional design was employed. Data were collected from 94 participants (34 teaching, 60 non‐teaching) via surveys and clinical measurements at the Kwame Nkrumah University of Science and Technology. Metabolic markers such as fasting blood glucose, cholesterol levels, abdominal obesity, and blood pressure were defined using the World Health Organisation cutoffs. Univariable logistic regression and multivariate models were adopted to analyse the association between lifestyle components and cardiometabolic markers.

**Results:**

Non‐teaching staff reported higher work stress (Job Content Questionnaire = 73.3%), while teaching staff showed lower physical activity (76.5%). Three dietary patterns were identified: Indigenous Ghanaian, Westernised, and High Protein. The most prevalent metabolic markers were high abdominal obesity (52.1%) and elevated blood pressure (19.1%). In multivariable logistic regression adjusting for age, education, income and staff category, high protein diet was associated with high blood pressure (aOR = 1.869, 95% CI = 1.011–3.455, *p* = 0.046).

**Conclusion:**

The results showed significant associations between some cardiometabolic markers and certain dietary patterns, while no significant association was found between work stress, physical activity, and cardiometabolic markers. The high prevalence of abdominal obesity and elevated blood pressure highlights the need for targeted workplace interventions. Based on border literature, workplace wellness programs aimed at reducing work stress and promoting physical activity while improving healthy diets will be beneficial to the staff.

## 1 Introduction

Metabolic markers are key predictors of cardiometabolic health and are strongly associated with increased cardiovascular disease, chronic kidney disease, and cognitive decline [1, 2]. These metabolic markers, including glucose levels and lipid profile, are influenced by lifestyle and environmental factors. Evidence from epidemiological studies highlights a substantial global burden of poor metabolic health. For example, a pooled analysis of thirty‐four studies including 26,609 young adults aged 18–30 years reported that 26.9–41.2% of participants exhibited low high‐density lipoprotein cholesterol [3].

In Ghana, a high prevalence of abnormal metabolic markers has been reported among occupational workers [4, 5]. Urban populations are particularly affected, potentially due to increased consumption of processed foods and reduced physical activity [6]. University employees, especially those in sedentary white‐collar roles, may be exposed to occupational and lifestyle‐related factors that could influence metabolic health [7]. In addition, chronic work stress, characterised by high demands and low control, has been associated with metabolic outcomes through neuroendocrine and inflammatory pathways [8, 9].

Although international studies support the association between occupational stress, poor diet, low physical activity, and metabolic health, few have investigated the relationship in the African university context. Many related studies have focused on market vendors, transport workers, and urban communities [4, 5], but the academic workplace, which is a high‐pressure environment with its lifestyle patterns, remains underexplored. This presents a critical gap as universities are increasingly recognised as a high‐risk environment for non‐communicable diseases [10]

This study aimed to explore the relationship between work stress, dietary habits, physical activity, and cardiometabolic markers among employees at the Kwame Nkrumah University of Science and Technology (KNUST). The findings are intended to generate evidence on potential lifestyle‐related determinants of cardiometabolic risk and highlight potential areas for further through larger population‐based studies. In this research, we hypothesized that occupational stress, dietary patterns, and physical activity (PA) may be associated with differences in cardiometabolic markers among university staff.

## 2 Materials and Methods

### 2.1 Participants and Sample Size

Data collection took place from 3 December 2024 to 25 June 2025. A total of 94 university workers were recruited from all six colleges at KNUST using a convenience sampling technique. The sample size was calculated using Cochran's formula, with 6% prevalence of metabolic syndrome 95% confidence level, and 5% error margin [11]. Although this calculation was used to guide recruitment for the prevalence objective of the study, no formal priori power calculation was conducted for the regression analyses. Therefore, regression findings should be interpreted as exploratory. This study employed a cross‐sectional design. Announcements were made to all workers to participate in this project. As the recruitment was based on voluntary response to these announcements, the total number of individuals who were exposed to the invitation, declined participation, or did not respond could not be ascertained. All eligible volunteers who consented during the data collection period were included in the study. Inclusion criteria: age ≥ 18 years and no diagnosis of mental disorders. Exclusion criteria: pregnant women and working years ≤ 1 year.

### 2.2 Demographic Questionnaire

A structured questionnaire was administered through in‐person interviews to collect socio‐demographic data. Primary details like gender, age, ethnic group, marital status, education level, health insurance coverage, salary, and work category (teaching and non‐teaching) were obtained.

### 2.3 Anthropometric and Clinical Measurements

Body weight was measured using an Omron scale (HBF‐514C, China) to the closest 0.1 kg without wearing any footwear. Height was measured using standard protocols with the use of a stadiometer. A Cotchear tape measure (China) was used to measure the waist circumference. When measuring the waist circumference, a horizontal line was drawn above the right hip bone while the participant stood. Blood pressure was measured using an Omron digital monitor (BP742N, Vietnam). Measurements were taken after a 5‐min rest in a seated position, with the arm supported at the heart level. The average of two readings was recorded.

### 2.4 Biochemical Analysis

Before data collection, the study participants were told to fast for 8 to 12 h, and about 5 millilitres of peripheral venous blood was collected. The blood samples were collected into clot activator tubes and transported on ice to the Clinical Analyses Laboratory (CAn‐Lab) to be processed for analyses. In the laboratory, blood samples underwent a centrifugation process, and the serum collected was used to check for lipid profile levels using a semi‐automatic KENZA BioChemisTry analyser (RM2030_18, France). Fasting blood glucose levels (FBG) were measured, and blood samples were collected between 7:00 am and 10:00 am. The left middle finger was pricked using sterile lancets to obtain a capillary blood sample to measure FBS with a Nocoding$1^{+}$ OnePlus (GM01BAA, Korea) glucometer.

### 2.5 Physical Activity and Dietary Pattern

For physical activity, the International Physical Activity Questionnaire (IPAQ) was used to measure the frequency and duration of physical activities in the past 7 days [12]. The total physical activity level was calculated and expressed in Metabolic Equivalent Task minutes per week (MET‐min/week) according to the IPAQ scoring protocol. Participants were then categorised into low, moderate, or high physical activity levels based on standard IPAQ classification criteria. For dietary patterns, a Food Frequency Questionnaire (FFQ) specifically designed for the Ghanaian population [13] was used to capture the frequency of commonly consumed foods over the past week. This questionnaire has been widely used in the adult population in Ghana [14, 15], however, validation studies specifically among university workers are limited, hence findings should be interpreted with caution. The questionnaire consisted of 60 individual food items. These items were subsequently aggregated into 14 predefined food groups based on similarities in nutritional composition. Principal Component Analysis (PCA) with Varimax rotation was conducted to extract major dietary patterns. Dietary patterns were named based on the food groups with the highest loadings to help interpretation, recognizing that these data driven patterns overlap and are not mutually exclusive. Sampling adequacy for factor analysis was confirmed using the Kaiser‐Meyer‐Olkin test (0.811) and Bartlett's test of sphericity (555.27, *p*< 0.001). However, the observation to variable ratio was 6.7 which is below the threshold of 10 and this may limit the stability of the identified patterns. Components were retained based on eigenvalues > 1 and scree plot evaluation. The dietary pattern distribution has been represented as a radar plotting (Supplementary Figure 1). Factor scores were divided into tertiles reflecting their level of contribution to each dietary pattern, with an assumed gradient from Tertile 1 (T1) to Tertile 3 (T3). T1 denoted low intake, T2 indicated medium intake, and T3 represented high intake of the respective pattern.

### 2.6 Work Stress Survey

Work‐related stress was measured using two validated instruments based on complementary theoretical models: the Job Content Questionnaire (JCQ) and the Effort‐Reward Imbalance (ERI) questionnaire. The JCQ (22 items) assessed psychological job demands, job control (decision latitude), and social support at work. It uniquely captures the imbalance between high job demands (e.g., workload intensity and task complexity) and low job control, with social support acting as a potential buffer. The ERI (23 items, scored on a 4‐point Likert scale) measured extrinsic effort, reward (including salary, esteem, job security, and career opportunities), and overcommitment. It uniquely evaluates the reciprocity between efforts expended and rewards received at work, as well as the individual's intrinsic overcommitment. These two instruments were selected because they assess distinct dimensions of work‐related stress. While the JCQ focuses primarily on the task characteristics of the job (demands *vs.* control and support), the ERI emphasizes the social exchange aspect (effort *vs.* rewards) and personal coping style (overcommitment). In the event of discordant findings between the two measures, results will be interpreted as reflecting different sources of stress: the JCQ may highlight stressful job task conditions that are nevertheless adequately rewarded, whereas the ERI may identify situations of insufficient reward of high overcommitment despite manageable demands and control. A ratio greater than 1 indicated high stress for both models.

ERI:Effort/Reward ratio=effort/(reward×6/11) [16]

JCQ:Demand/Control ratio=Job demand/(job control×5/9) [17].

Internal reliability was confirmed via Cronbach's alpha: 0.693 for JCQ and 0.519 for ERI. Although the ERI Cronbach's alpha falls below the accepted threshold of 0.70, the scale was retained due to its complementary contribution to the JCQ. Therefore, the ERI findings may not be the definite nature of stress at the university. ERI has previously been used in a similar population in Nigeria [18].

### 2.7 Cardiometabolic Markers

The cutoffs for cardiometabolic markers according to the World Health Organisation were used:

1. Waist circumference (cm): Males > 94 and Females > 80
2. High density lipoprotein (HDL) cholesterol (mmol/L): Males < 1.03 and Females < 1.29
3. Triglycerides (mmol/L): ≥ 1.7
4. Fasting glucose (mmol/L): ≥ 6.1
5. Blood pressure (mmHg): ≥ 140/90

### 2.8 Ethical Approval

This study was conducted according to the guidelines laid down in the Declaration of Helsinki, and all procedures involving research study participants were approved by the KNUST Committee on Human Research, Publication and Ethics (CHRPE/AP/1218/24). Written informed consent was obtained from all subjects.

### 2.9 Data Analysis

Data analysis was carried out using Statistical Package for the Social Sciences (SPSS) version 24. Chi‐square tests were used to determine the differences between teaching staff and non‐teaching staff, whereas independent t‐tests were used to compare means and standard deviations. Univariate regression analysis was performed to determine the association between work stress, physical activity and cardiometabolic markers (Table 4). It was also used to determine the association between dietary patterns, stress, physical activity and cardiometabolic markers (Table 5). Multivariate regression analysis was further conducted to determine the influence of stress, dietary patterns, physical activity and cardiometabolic markers (Table 5 and Supplementary Table 3). With the supplementary table 3, sex, education, income and age were adjusted in the analysis. All statistical tests were two‐sided, and a *p*‐value of less than 0.05 was considered statistically significant.

## 3 Results

The sample consisted of 94 participants (60 non‐teaching staff, 34 teaching staff). Majority of the staff were between the ages of 35 to 44 (teaching staff; 19 and non‐teaching staff: 15). The teaching staff were predominantly males [30] whereas most of the non‐teaching staff were females [44]. All the teaching staff had tertiary education (100%) while 45.0% (27/60) of the non‐teaching staff had primary education. No significant p‐value was found in health insurance status (*p *= 0.132) or number of children (*p *= 0.161). The *p* values were obtained using Chi‐square test. Detailed sociodemographic characteristics are provided in Supplementary Table 1.

Table 1 shows that teaching staff had much lower PA levels than non‐teaching staff (*p *< 0.001), with more than three‐quarters (76.5%; 26/34) of them being classified as low active, compared to 30.0% (18/60) of non‐teaching staff. The ERI scores for work stress did not show any statistical difference in stress levels (*p* = 0.410) but the JCQ scores showed significant statistical difference in the stress levels between teaching and non‐teaching staff (*p *< 0.001). About 94.1% (32/34) of teaching staff had low job strain, while only 26.7% (16/60) of non‐teaching staff did (*p *< 0.001). The *p* values were obtained using Chi‐square test. The JCQ measure showed that non‐teaching staff were more stressed.

**Table 1:** ERI, JCQ, and Physical Activity Assessment of Work Stress Among Teaching and Non‐Teaching Staff.

Variable	Category	Teaching staff *N* (%)	Non‐teaching staff *N* (%)	Total	*p*‐value
ERI	Low	20 (58.8%)	30 (50.0%)	50 (53.2%)	0.410
	High	14 (41.2%)	30 (50.0%)	44 (46.8%)	
	Mean	0.97	1.00	0.99	0.735
	SD	0.38	0.28	0.32	
JCQ	Low	32 (94.1%)	16 (26.7%)	48 (51.1%)	< 0.001
	High	2 (5.9%)	44 (73.3%)	46 (48.9%)	
	Mean	0.83	1.11	1.01	< 0.001
	SD	0.13	0.20	0.22	
PA	Low	26 (76.5%)	18 (30.0%)	44 (46.8%)	< 0.001
	Moderate	8 (23.5%)	37 (61.7%)	45 (47.9%)	
	High	0 (0.0%)	5 (8.3%)	5 (5.3%)	
	Mean	1.24	1.78	1.59	< 0.001
	SD	0.43	0.58	0.59	

*Note:* Independent t‐tests were used to compare means of continuous variables between groups whereas categorical variables were compared using Chi‐square tests (*p*‐values).

Abbreviations: ERI = Effort Reward Imbalance, JCQ = Job Content Questionnaire, PA = Physical Activity, SD = Standard Deviation.

Table 2 illustrates the significant differences in dietary patterns consumed by teaching and non‐teaching staff. Three dietary patterns were identified using factor analysis: Indigenous Ghanaian Diet, Westernised Diet and High Protein Diet (see Supplementary Table 2). Regarding the indigenous Ghanaian diet, no significant differences were observed (*p* = 0.706). Teaching staff were primarily high‐level consumers of the Westernised Diet, whereas non‐teaching staff were more likely to consume that at a low level (*p *< 0.001). The High Protein Diet also showed significant differences (*p* = 0.009), with teaching staff exhibiting higher consumption patterns than non‐teaching staff. The *p* values were obtained using Chi‐square test.

**Table 2:** Consumption Level of Dietary Patterns Between Teaching Staff and Non‐Teaching Staff.

Dietary pattern	Level of consumption	Teaching staff	Non‐teaching staff	Total	*p*‐value
Indigenous Ghanaian diet	Low	13 (38.2%)	18 (30.0%)	31 (33.0%)	0.706
	Medium	11 (32.4%)	21 (35.0%)	32 (34.0%)	
	High	10 (29.4%)	21 (35.0%)	31 (33.0%)	
Westernised diet	Low	7 (20.6%)	24 (40.0%)	31 (33.0%)	< 0.001
	Medium	7 (20.6%)	25 (41.7%)	32 (34.0%)	
	High	20 (58.8%)	11 (18.3%)	31 (33.0%)	
High protein diet	Low	5 (14.7%)	26 (43.3%)	31 (33.0%)	0.009
	Medium	17 (50.0%)	15 (25.0%)	32 (34.0%)	
	High	12 (35.3%)	19 (31.7%)	31 (33.0%)	

*Note: p*‐values were obtained using the Chi‐square test.

Table 3 summarises the prevalence of cardiometabolic markers among the university staff. The most prevalent components were abdominal obesity (52.1%) and elevated blood pressure (19.1%). More than half (53.3%) of the non‐teaching staff had abnormal abdominal obesity while 50% of the teaching staff had abnormal abdominal obesity. Elevated fasting glucose was the least common (11.7%) cardiometabolic marker. No statistically significant differences were found between teaching and non‐teaching staff for any metabolic marker (all *p* > 0.05). The *p* values were obtained using Chi‐square test.

**Table 3:** Prevalence of Cardiometabolic Markers Among University Staff.

Cardiometabolic markers	Teaching staff *N* = 34 *N* (%)	Non‐teaching staff *N* = 60 *N* (%)	Total	*p*‐values
Abdominal obesity	17 (50.0%)	32 (53.3%)	49 (52.1%)	0.759
Elevated triglycerides	8 (23.5%)	6 (10.0%)	14 (14.9%)	0.077
Reduced HDL cholesterol	0 (0.0%)	0 (0.0%)	0 (0.0%)	—
Elevated blood pressure	8 (23.5%)	10 (16.7%)	18 (19.1%)	0.422
Elevated fasting glucose	6 (17.6%)	5 (8.3%)	11 (11.7%)	0.223

*Note: p*‐values were obtained using the Chi‐square test. The mean and standard deviation for HDL (94 participants) was 1.85 ± 0.21.

As shown in Table 4, the univariate regression analysis did not show significant associations between stress, physical activity and cardiometabolic markers. All *p* values were > 0.05.

**Table 4:** Univariate Associations Between Work Stress, Physical Activity, and Cardiometabolic Markers.

Cardiometabolic markers	Work stress		Physical activity
	ERI OR (95% CI) *p*‐value	JCQ OR (95% CI) *p*‐value	OR (95% CI) *p*‐value
Elevated fasting glucose	0.493 (0.063–3.880) 0.502	0.075 (0.003–1.862) 0.114	1.000 (0.999–1.000) 0.696
Abdominal obesity	0.799 (0.223–2.861) 0.730	4.197 (0.612–28.781) 0.144	1.000 (1.000–1.000) 0.520
High triglycerides	1.495 (0.258–8.665) 0.654	0.054 (0.003–1.044) 0.053	0.999 (0.998–1.000) 0.082
High blood pressure	2.300 (0.469–11.293) 0.305	0.497 (0.045–5.475) 0.568	1.000 (0.999–1.000) 0.580

Abbreviations: CI, confidence interval; ERI, effort reward imbalance; JCQ, job content questionnaire; OR, odds ratio.

In Table 5, the multivariate regression analysis showed significant association between blood pressure and high protein diet. After adjusting (Supplementary Table 3) for sex, age, education and income, there was a significant association between high blood pressure and high protein diet (aOR = 1.869, CI = 1.011–3.455, *p* = 0.046). A significant association was also observed between abdominal obesity and westernised diet (OR = 0.547, 95% CI = 0.330–0.906, *p* = 0.019; aOR = 0.357, 95% CI = 0.165–0.771, *p* = 0.009).

**Table 5:** Multivariate associations between lifestyle factors and cardiometabolic markers.

Cardiometabolic markers	PA aOR (95% CI) *p*‐value	Indigenous aOR (95% CI) *p*‐value	Westernized aOR (95% CI) *p*‐value	High protein aOR (95% CI) *p*‐value	JCQ aOR (95% CI) *p*‐value	ERI OR (95% CI) *p*‐value
FBS	1.000 (1.000–1.000) 0.747	1.704 (0.854–3.400) 0.131	0.591 (0.269–1.295) 0.189	1.145 (0.534–2.457) 0.728	0.025 (0.001–1.078) 0.055	0.149 (0.012–1.893) 0.142
Blood pressure	1.000 (0.999–1.000) 0.660	1.308 (0.773–2.215) 0.318	0.836 (0.462–1.513) 0.554	1.623 (0.928–2.837) 0.090	0.561 (0.033–9.608) 0.690	2.332 (0.418–13.012) 0.335
Abdominal obesity	1.000 (1.000–1.000) 0.585	0.828 (0.521–1.316) 0.425	0.547 (0.330–0.906) 0.019	1.187 (0.764–1.844) 0.446	2.224 (0.245–20.214) 0.478	0.736 (0.174–3.108) 0.677
High triglycerides	0.999 (0.998–1.000) 0.155	1.568 (0.814–3.021) 0.179	0.625 (0.306–1.277) 0.197	0.686 (0.346–1.359) 0.280	0.070 (0.003–1.821) 0.110	1.552 (0.212–11.363) 0.665

## 4 Discussion

This study investigated the relationship between dietary patterns, PA, work stress, and metabolic markers among university workers in Ghana. The results showed that there were significant differences in jobs and lifestyles between the teaching and non‐teaching staff. Work stress was higher among non‐teaching staff according to the Job Content Questionnaire. Teaching staff exhibited lower physical activity reflecting sedentary roles. There was a significant association between high protein diet and high blood pressure after adjusting for age, sex, income and educational background. Abdominal obesity was the most prevalent metabolic marker.

Work stress findings reveal a varied outcome. The JCQ indicated significantly higher stress among non‐teaching staff than the teaching staff. This difference likely stems from non‐teaching roles, such as administrative or manual tasks, which offer less autonomy and more repetitive demands. This eliminates job control, which is a key JCQ component. Teaching staff, while facing pressures from research and lecturing, may have greater flexibility, mitigating perceived stress. The detection rates of occupational stress in Job Demand‐Control (JDC) and Effort‐Reward Imbalance (ERI) models in petrochemical workers in China were 28.4% and 27.2%, respectively [19], which are lower than the JCQ findings in this study, possibly reflecting differences in occupational contexts or assessment tools. A study in Nigeria that utilized the Effort Reward Imbalance questionnaire observed that 62.20% are highly committed, and 33.44% are moderately committed, while 8.62% were moderately stressed and 91.38% were highly stressed [18]. This suggests a cultural context where high commitment may coexist with elevated stress levels. These findings resonate with a study by Kodua‐Ntim et al. (2021) on library staff in Ghanaian universities reported high stress due to workload and inadequate resources [20]. This pattern is likely applicable to non‐teaching staff in this study. Similarly, Wickramasinghe (2016) noted that administrative roles in the public sector often lack clear promotional pathways contributing to stress [21]. The higher stress among non‐teaching staff may also reflect socioeconomic vulnerabilities, as lower income and education levels (see Supplementary Table 1), prevalent in this group, are linked to psychological strain [22]. The non‐significant ERI result might indicate a cultural normalisation of effort‐reward imbalances in Ghana, where long hours are often expected, particularly in public institutions.

Physical activity levels highlighted an important distribution, with teaching staff exhibiting lower engagement compared to non‐teaching staff. None of the teaching staff reported high physical activity whereas 8.3% (5/60) of non‐teaching staff reported high physical activity. The sedentary nature of academic work, including lectures, meetings, and research, likely contributes to low activity among teaching staff, a trend observed among Ghanaian academics by Keenan et al. (2015) [23]. Non‐teaching staff, engaged in more physically demanding tasks like cleaning or campus mobility, reported higher moderate and high activity levels, reflecting occupational differences. This trend is consistent with research from Africa showing that people in cities tend to be more inactive as they move into higher‐ranking jobs [24]. Research shows that education and age influence physical activity, with higher‐educated groups like teaching staff often less active due to desk‐based roles [25]. The majority of teaching staff from primary schools in the Cape Coast metropolis were physically inactive [26], a finding that is similar to the sedentary trends among teaching staff in this study. Culturally, time constraints and a work‐centric mindset in Ghana may discourage exercise, particularly among teaching staff, as noted in urban professional cohort [27]. The low activity levels, especially among teaching staff, signal a risk for non‐communicable diseases (NCDs), highlighting the need for workplace interventions to promote movement.

Three major dietary patterns were identified amongst the cohort (see Supplementary Table 2). The prominence of indigenous foods reflects cultural persistence, as noted by Miller (2021) [28], who found traditional staples dominate Ghanaian diets despite urbanisation as seen in this study. The Westernised pattern which was common among younger staff indicates a nutrition transition driven by globalisation and urban access to processed foods, a trend documented by Nel and Steyn (2022) [29]. Urban living often involves fast‐paced work routines, leading to greater dependence on prepared, processed, pre‐packaged, and ready‐to‐eat foods [30], which may contribute to the adoption of the Westernised pattern. A study by Kroll et al. (2019) confirmed the high level of purchase and consumption of ultra‐processed foods in urban South Africa and Ghana [31], which has been implicated in the rising levels of obesity among urban dwellers. These patterns may reflect socioeconomic influences. Teaching staff with higher incomes may have greater access to Westernised foods, while non‐teaching staff with lower earnings may rely on cost‐effective indigenous diets. A study in urban Ghana identified similar mixed dietary patterns, with education and income (Table 2; Supplementary Table 1) driving Western food adoption [32]. The increasing levels of obesity have also been attributed to changes in lifestyle behaviours, including the overconsumption of highly processed food and less intake of fruits and vegetables [4], which may influence the dietary transition observed. The high‐protein diet suggests some staff, possibly health‐conscious or higher‐earning, prioritise nutrient‐dense foods, aligning with shifts toward protein consumption in urban Ghana [33]. The interplay of tradition and modernity in staff diets highlights the complexity of addressing nutritional health in Ghanaian workplaces, where cultural preferences coexist with global influences. Although the analysis yielded three interpretable components and was supported by acceptable KMO and Bartlett's test values, the cross‐loadings observed in Table 2 suggest that the derived patterns may not be fully stable. A larger sample size could yield a different factor structure. Therefore, future studies with larger samples are needed to confirm the stability of these findings.

In this study, abdominal obesity (52.1%; 49/94) and elevated blood pressure (19.1%; 18/94) were the most common metabolic markers. The higher prevalence of some cardiometabolic markers among the staff should be interpreted with caution given the sex imbalance between groups and potential confounding by sex. These findings align with national trends, where central adiposity and hypertension are rising among urban dwellers [34]. This shows a cardiometabolic burden among this population. Research indicates that elevated blood pressure causes arteriolar narrowing, thickening, and leakage in retinal vessels, resulting in hypertensive retinopathy, while excess adipose tissue contributes to chronic kidney disease through its endocrine activity and persistent low‐grade inflammation [35, 36]. The absence of reduced HDL cholesterol in this study is notable, as low HDL cholesterol is commonly reported in metabolic syndrome populations. This finding is reported as observed in the study population. No additional explanatory data was available to further interpret this result. Further studies are needed to confirm this finding of this population. Univariate analysis showed no significant relationship between metabolic markers and lifestyle factors (physical activity and stress, all *p* > 0.05). This result aligns with findings from Iran, where no significant relationship existed between occupational stress and risk factors of cardiovascular disease [37], which further supports the complexity of stress and metabolic markers in this population. Lifestyle‐related factors such as physical activity and work stress have been well‐documented to predispose individuals to metabolic risk [19]. However, our current study did not conform to findings in the literature which highlight the need for further investigation. No causal inference can be drawn from the inverse association between westernised pattern and abdominal obesity. This is not a true protective effect of westernised dietary intake but can rather be the composition of the pattern. Therefore, this result should be interpreted with caution and validated using continuous dietary scores. In Mexican adults, a Westernised dietary pattern was positively associated with both overweight‐obesity and abdominal obesity compared with a rural pattern [38].

In our study, significant association was observed between high protein diet consumption and high blood pressure after adjusting for age, sex, education and income. While this finding suggests a possible relationship in the Ghanaian context, it should be interpreted cautiously given the cross‐sectional design and exploratory nature. The aggregation of the 60 food items into 14 also reduces dietary granularity, particularly for sodium‐rich food sources which can affect this observed association. In addition, the wide confidence interval observed in some associations indicates imprecision and a further study will be required to better understand how high protein can contribute to hypertension among Ghanaian populations. This result differs from some evidence suggesting protective effects of protein‐rich diets [39]. Previous studies have suggested that high intake of processed or salted protein‐rich foods may be associated with elevated blood pressure; however, such mechanisms cannot be inferred from the present data [40, 41, 42]. Overall, this finding should be considered hypothesis generating and requires confirmation in longitudinal studies.

### 4.1 Strengths and Limitations of the Study

This research is relevant to public health as it addresses a growing concern, thus non‐communicable diseases in a specific understudied population. It combines lifestyle, occupational stress and biomarkers which gives a holistic view of the situation. This study employs a multi‐dimensional approach integrating psychosocial (stress), behavioral (diet and physical activity) and clinical (biomarkers) dimensions. The study also utilizes validated tools like the IPAQ and two stress questions the JCQ and the ERI. Further, focusing on Ghanaian university workers provides context‐specific evidence that is valuable for local health policy and workplace intervention, and the results of the study can be adapted in populations with similar conditions. This research has a huge potential for impact as it can inform workplace wellness programs, university health promotion policies and national NCD prevention strategies. This study also has several limitations. The cross‐sectional design and the use of convenient sampling preclude causal inferences, limiting understanding of how stress, activity, or diet influences cardiometabolic markers over time. This can also underestimate or overestimate prevalence of cardiometabolic markers and lifestyle behaviours. Self‐reported data for PA and dietary patterns introduce recall bias, which could potentially affect the reliability of the results. The single‐institution sample restricts generalisability to other Ghanaian universities, where staff profiles may differ. The moderate sample size (*N* = 94) may also limit statistical power for detecting subtle associations. Additionally, unmeasured covariates such as genetic predisposition, sleep quality, or psychosocial factors may influence cardiometabolic markers. The long duration of the data collection could also affect the variability of the data due to seasonal changes in diet and physical activity. Capillary blood used for the fasting glucose measurement and the sex imbalance between the staff are also limitations.

## 5 Conclusion

This research provides a detailed narrative of the health and lifestyle of Ghanaian university staff which uncovers occupational disparities that reflect wider socioeconomic trends. Teaching staff experience sedentary lifestyles, whereas non‐teaching staff encounter high stress. The results showed that there was a significant association between high protein consumption with high blood pressure after adjusting the multivariate model. Our results provide a scientific basis for further research to assess how lifestyle factors affect cardiometabolic health among Ghanaians, especially high protein diet and high blood pressure.

### 5.1 Future Directions

Subsequent research should use longitudinal methodologies to investigate causal relationships between occupational stress, physical activity, dietary behaviour, and metabolic risk. Broadening research to encompass various Ghanaian universities would augment external validity, facilitating the capture of regional variations. Accelerometers can be used for physical activity assessment and dietary biomarkers for nutrient intake assessment which can improve data reliability and address self‐report biases. Subsequent studies can also investigate additional covariates such as sleep or genetic factors to provide a holistic view of metabolic risk.

## Author Contributions

**Daniel Ayeltigah:** methodology, formal analysis, writing – original draft, writing – review and editing, project administration, data curation. **Mary Amoako:** conceptualization, methodology, formal analysis, writing – review and editing, supervision. **Richard Oguah:** methodology, writing – review and editing, data curation. **Dorcas Kafui Ledi:** methodology, writing – review and editing, data curation. **Adwoa Gyamfi:** supervision, writing – review and editing. **Michael Akenteng Wiafe:** supervision, writing – review and editing.

## Funding

The authors have nothing to report.

## AI Disclosure

The authors declare that no artificial intelligence (AI) tools or AI‐assisted technologies were used in the preparation of this manuscript, data analysis, or generation of figures.

## Conflicts of Interest

The authors declare no conflicts of interest.

## Transparency Statement

I Daniel Ayeltigah affirms that this manuscript is an honest, accurate, and transparent account of the study being reported; that no important aspects of the study have been omitted; and that any discrepancies from the study as planned (and, if relevant, registered) have been explained.

## Supporting information


Supporting File

## Data Availability

The data that support the findings of this study are available on request from the corresponding author. The data are not publicly available due to privacy or ethical restrictions. The data supporting the findings of this study are not publicly available due to ethical restrictions. However, the data may be made available from the corresponding author upon reasonable request and subject to approval by the Kwame Nkrumah University of Science and Technology Committee on Human Research, Publication, and Ethics.

## References

[1] M. Tahmi , P. Palta , and J. A. Luchsinger , “Metabolic Syndrome and Cognitive Function,” Current Cardiology Reports 23, no. 12 (2021): 180. 10.1007/s11886-021-01615-y. 34668083

[2] T. Joyce , Y. I. Chirino , M. T. Natalia , and P. C. Jose , “Renal Damage in the Metabolic Syndrome (MetSx): Disorders Implicated,” European Journal of Pharmacology 818 (2018): 554–568, 10.1016/j.ejphar.2017.11.032. 29162432

[3] P. B. Nolan , G. Carrick‐Ranson , J. W. Stinear , S. A. Reading , and L. C. Dalleck , “Prevalence of Metabolic Syndrome and Metabolic Syndrome Components in Young Adults: A Pooled Analysis,” Preventive Medicine Reports 7 (2017): 211–215, 10.1016/j.pmedr.2017.07.004. 28794957 PMC5540707

[4] C. Apprey , B. A. Baah‐Nuako , V. T. Annaful , A. O. Adebanji , and V. Dzogbefia , “Dietary Intake and Prevalence of Metabolic Syndrome Among Tanker Truck Drivers in Ghana,” Nutrition & Food Science 52, no. 7 (2022): 1055–1069, 10.1108/NFS-08-2021-0250.

[5] C. A. Appiah , E. O. Afriyie , F. E. A. Hayford , and E. Frimpong , “Prevalence and Lifestyle‐Associated Risk Factors of Metabolic Syndrome Among Commercial Motor Vehicle Drivers in a Metropolitan City in Ghana,” Pan African Medical Journal 36 (2020): 136. 10.11604/pamj.2020.36.136.16861. 32849991 PMC7422739

[6] F. Agbozo , E. Bannerman , S. Klomegah , and F. Zotor , “Lifestyle Habits and Perceived Wellbeing of Adults Presenting With Metabolic Syndrome at a Diabetic Clinic in Ghana: A Case‐Control Study,” Human Nutrition & Metabolism 29 (2022): 200154, 10.1016/j.hnm.2022.200154.

[7] L. Rahma Listyandini , P. Fenti Dewi Pertiwi , R. Dian Puspa Riana , and Widya Asih Lestari , “The Dominant Factor of Metabolic Syndrome Among Office Workers,” Journal of Health Science and Prevention 5, no. 1 (2021): 40–48, 10.29080/jhsp.v5i1.421.

[8] D. Ratih Damayanti , W. Ratnaningtyas Wahyu Kusuma Wardhani , P. Berliana Devianti Putri , and Indah Lutfiya , “The Job Stress, Workload, Exercise Habits and Metabolic Syndrome in Academic Personnel at Airlangga University,” Malaysian Journal of Public Health Medicine 20, no. 2 (2020): 276–284, 10.37268/mjphm/vol.20/no.2/art.403.

[9] T. Gerding and J. Wang , “Stressed at Work: Investigating the Relationship between Occupational Stress and Salivary Cortisol Fluctuations,” International Journal of Environmental Research and Public Health 19, no. 19 (2022): 12311, 10.3390/ijerph191912311. 36231612 PMC9564551

[10] D. Bunsong , M. Chatchumni , U. Kaewsuk , and P. Boonkrong , “Health Promotion Research at Rangsit University: A Scoping Review Using the HURS Framework for Institutional Policy Development,” Journal of Current Science and Technology 16, no. 1 (2025): 160, 10.59796/jcst.V16N1.2026.160.

[11] R. Ofori‐Asenso , A. A. Agyeman , and A. Laar , “Metabolic Syndrome in Apparently “Healthy” Ghanaian Adults: A Systematic Review and Meta‐Analysis,” International Journal of Chronic Diseases 2017 (2017): 1–9, 10.1155/2017/2562374. PMC5654269 29130065

[12] C. L. Craig , A. L. Marshall , M. Sjöström , et al., “International Physical Activity Questionnaire: 12‐Country Reliability and Validity,” Medicine and Science in Sports and Exercise 35, no. 8 (2003): 1381–1395, 10.1249/01.MSS.0000078924.61453.FB. 12900694

[13] F. A. A. P. Essiam , M. Amoako , E. K. Owusu , et al., “Intersecting Burdens: Oral Health, Dietary Patterns, Food Security and Their Impact on Cardiometabolic Risk and Mental Health in Ghana,” BMC Nutrition 11, no. 1 (2025): 228. 10.1186/s40795-025-01223-x. 41419975 PMC12750599

[14] S. A. Tonyemevor , M. Amoako , L. J. J. Gowans , et al., “Maternal Dietary Patterns, Food Security and Multivitamin Use as Determinants of Non‐Syndromic Orofacial Clefts Risk in Ghana: A Case–Control Study,” Women 5, no. 3 (2025): 34, 10.3390/women5030034.

[15] D. Ayeltigah , N. G. Wuabu , M. Amoako , M. O. Amoako , and C. A. Appiah , “Work Stress, Dietary Patterns, and Physical Activity on Metabolic and Hepatic Biomarkers Among Healthcare Professionals in Ghana,” Journal of Health, Population, and Nutrition 45 (2026): 173. 10.1186/s41043-026-01344-4. 42152142 PMC13386725

[16] R. A. Karasek , “Job Demands, Job Decision Latitude, and Mental Strain: Implications for Job Redesign,” Administrative Science Quarterly 24, no. 2 (1979): 285. 10.2307/2392498.

[17] J. Siegrist , D. Starke , T. Chandola , et al., “The Measurement of Effort‐Reward Imbalance at Work: European Comparisons,” Social Science & Medicine (1982) 58, no. 8 (2004): 1483–1499. 14759692 10.1016/S0277-9536(03)00351-4

[18] A. Asouzu and N. Umerah , “P02‐002‐23 Work Stress and Metabolic Syndrome Among Tertiary Institution Workers in Enugu Metropolis, Nigeria,” Current Developments in Nutrition 7 (2023): 100233, 10.1016/j.cdnut.2023.100233.

[19] M. Zhang , B. Liu , W. Ke , et al., “Correlation Analysis between Occupational Stress and Metabolic Syndrome in Workers of a Petrochemical Enterprise: Based on Two Assessment Models of Occupational Stress,” BMC Public Health 24, no. 1 (2024): 802. 10.1186/s12889-024-18305-3. 38486274 PMC10938751

[20] K. Kodua‐Ntim , H. Akussah , and E. Adjei , “Managing Stress Among Library Staff in Public University Libraries in Ghana,” The Journal of Academic Librarianship 47, no. 4 (2021): 102362, 10.1016/j.acalib.2021.102362.

[21] V. Wickramasinghe , “The Mediating Effect of Job Stress in the Relationship between Work‐Related Dimensions and Career Commitment,” Journal of Health Organization and Management 30, no. 3 (2016): 408–420, 10.1108/JHOM-06-2014-0094. 27119394

[22] G. Navarro‐Carrillo , M. Alonso‐Ferres , M. Moya , and I. Valor‐Segura , “Socioeconomic Status and Psychological Well‐Being: Revisiting the Role of Subjective Socioeconomic Status,” Frontiers in Psychology 11 (2020): 1303, 10.3389/fpsyg.2020.01303. 32587560 PMC7298147

[23] M. Keenan and A. E. Greer , “Sedentary Behavior and Related Factors Among Full‐Time, University Faculty,” International Journal of Workplace Health Management 8, no. 3 (2015): 206–213, 10.1108/IJWHM-09-2014-0034.

[24] L. K. Micklesfield , K. Westgate , A. Smith , et al., “Physical Activity and Posture Profile of a South African Cohort of Middle‐Aged Men and Women as Determined by Integrated Hip and Thigh Accelerometry,” Epidemiology 1 (2021): 21265362. http://medrxiv.org/lookup/doi/10.1101/2021.10.22.21265362.

[25] C. Bertuol , A. V. B. Tozetto , S. N. De Oliveira , and G. F. Del Duca , “Sex Differences in the Association between Educational Level and Specific Domains of Physical Activity: A Brazilian Cross‐National Survey,” Canadian Journal of Public Health 113, no. 3 (2022): 474–483, 10.17269/s41997-021-00594-5. 34988924 PMC8731185

[26] E. W. Ansah , M. Adabla , N. Jerry , E. A. Aloko , and J. E. Hagan , “Investigating Sedentariness and Health Status of Primary School Teachers in Ghana,” BMC Health Services Research 23, no. 1 (2023): 983. 10.1186/s12913-023-09925-3. 37700305 PMC10498583

[27] G. B. Nketiah , K. Odoi‐Agyarko , T. A. Ndanu , et al., “Physical Inactivity Among Corporate Bank Workers in Accra, Ghana: Implications for Health Promotion,” PLoS One 18, no. 5 (2023): e0277994, 10.1371/journal.pone.0277994. 37167293 PMC10174574

[28] B. Simpson Miller , “Gold Coast Foodways in the Nineteenth Century.” Food and Identity in Nineteenth and Twentieth Century Ghana [Internet] (Cham: Springer International Publishing, 2021), 143–178. (Food and Identity in a Globalising World). Available from. doi:1 0.1007/978‐3‐030‐88403‐1_5 https://link.springer.com/10.1007/978-3-030-88403-1_5.

[29] J. H. Nel and N. P. Steyn , “The Nutrition Transition and the Double Burden of Malnutrition in Sub‐Saharan African Countries: How Do These Countries Compare With the Recommended Lancet Commission Global Diet?,” International Journal of Environmental Research and Public Health 19, no. 24 (2022): 16791, 10.3390/ijerph192416791. 36554669 PMC9779835

[30] M. Holdsworth , R. Pradeilles , A. Tandoh , et al., “Unhealthy Eating Practices of City‐Dwelling Africans in Deprived Neighbourhoods: Evidence for Policy Action From Ghana and Kenya,” Global Food Security 26 (2020): 100452, 10.1016/j.gfs.2020.100452. 33324537 PMC7726234

[31] F. Kroll , E. C. Swart , R. A. Annan , et al., “Mapping Obesogenic Food Environments in South Africa and Ghana: Correlations and Contradictions,” Sustainability 11, no. 14 (2019): 3924, 10.3390/su11143924.

[32] S. B. Kushitor , D. O. Alangea , R. Aryeetey , et al., “Dietary Patterns Among Adults in Three Low‐Income Urban Communities in Accra, Ghana,” PLoS One 18, no. 11 (2023): e0293726, 10.1371/journal.pone.0293726. 37943866 PMC10635542

[33] C. L. Mingle , G. Darko , N. K. Asare‐Donkor , L. S. Borquaye , and E. Woode , “Patterns in Protein Consumption in Ghanaian Cities,” Scientific African 11 (2021): e00684, 10.1016/j.sciaf.2020.e00684.

[34] M. T. Yussif , A. E. Morrison , and R. A. Annan , “10‐year Level, Trends and Socio‐Demographic Disparities of Obesity Among Ghanaian Adults—A Systematic Review and Meta‐Analysis of Observational Studies,” PLOS Glob Public Health 4, no. 1 (2024): e0002844, 10.1371/journal.pgph.0002844. 38271466 PMC10810441

[35] T. Arabi , A. Shafqat , B. N. Sabbah , et al., “Obesity‐Related Kidney Disease: Beyond Hypertension and Insulin‐Resistance,” Frontiers in Endocrinology 13 (2023): 1095211, 10.3389/fendo.2022.1095211. 36726470 PMC9884830

[36] E. Di Marco , F. Aiello , M. Lombardo , et al., “A Literature Review of Hypertensive Retinopathy: Systemic Correlations and New Technologies,” European Review for Medical and Pharmacological Sciences 26, no. 18 (2022): 6424–6443, 10.26355/eurrev_202209_29742. 36196693

[37] A. Shahbazi , N. Rahmani , M. Abbasi , et al., “Association between Occupational Stress and Risk Factors of Cardiovascular Disease in Locomotive Operators,” Iranian Heart Journal 19, no. 2 (2018): 20–26.

[38] S. Rodríguez‐Ramírez , B. Martinez‐Tapia , D. González‐Castell , L. Cuevas‐Nasu , and T. Shamah‐Levy , “Westernized and Diverse Dietary Patterns Are Associated With Overweight‐Obesity and Abdominal Obesity in Mexican Adult Men,” Frontiers in Nutrition 9 (2022): 891609, 10.3389/fnut.2022.891609. 35811984 PMC9263742

[39] J. J. Lim , Y. Liu , L. W. Lu , D. Barnett , I. R. Sequeira , and S. D. Poppitt , “Does a Higher Protein Diet Promote Satiety and Weight Loss Independent of Carbohydrate Content? An 8‐Week Low‐Energy Diet (LED) Intervention,” Nutrients 14, no. 3 (2022): 538, 10.3390/nu14030538. 35276894 PMC8838013

[40] A. S. Oyekale , “Effect of Obesity and Other Risk Factors on Hypertension Among Women of Reproductive Age in Ghana: An Instrumental Variable Probit Model,” International Journal of Environmental Research and Public Health 16, no. 23 (2019): 4699, 10.3390/ijerph16234699. 31779087 PMC6926784

[41] M. I. F. Mendes , R. D. Mendonça , C. M. O. Aprelini , and M. C. B. Molina , “Consumption of Processed Meat but Not Red Meat Is Associated With the Incidence of Hypertension: ELSA‐Brasil Cohort,” Nutrition 127 (2024): 112529, 10.1016/j.nut.2024.112529. 39154548

[42] Y. Zhang and D. Zhang , “Red Meat, Poultry, and Egg Consumption With the Risk of Hypertension: A Meta‐Analysis of Prospective Cohort Studies,” Journal of Human Hypertension 32, no. 7 (2018): 507–517, 10.1038/s41371-018-0068-8. 29725070

