# Supplementary material for: Association Between Dietary Patterns, Work Stress, Physical Activity, and Cardiometabolic Markers Among University Workers in Ghana: A Cross‐Sectional Study
Source: Health Sci Rep. 2026 Aug 5;9(8):e72986. doi: 10.1002/hsr2.72986 (PMC13443126; doi:10.1002/hsr2.72986)
Supplement: Supplementary file 1 [file HSR2-9-e72986-s001.docx]

**Supplementary Table 1. Sociodemographic Characteristics of University staff by Work Category**

| **Variable** | **Category** | **Teaching staff**  **N (%)** | **Non-teaching staff**  **N (%)** | **P-values** |
| --- | --- | --- | --- | --- |
| Age Group of Participants | 18-24 | 0 (0.0%) | 5 (8.3%) | 0.015 |
|  | 25-34 | 4 (11.8%) | 13 (21.7%) |  |
|  | 35-44 | 19 (55.9%) | 15 (25.0%) |  |
|  | 45-54 | 3 (8.8%) | 14 (23.3%) |  |
|  | 55 and above | 8 (23.5%) | 13 (21.7%) |  |
| Sex | Male | 30 (88.2%) | 16 (26.7%) | < .001 |
|  | Female | 4 (11.8%) | 44 (73.3%) |  |
| Highest Level of Education | No formal education | 0 (0.0%) | 11 (18.3%) | < .001 |
|  | Primary | 0 (0.0%) | 27 (45.0%) |  |
|  | Secondary | 0 (0.0%) | 9 (15.0%) |  |
|  | Tertiary | 34 (100.0%) | 13 (21.7%) |  |
| Monthly Income | Under 900 | 0 (0.0%) | 43 (71.7%) | < .001 |
|  | 1000-2900 | 0 (0.0%) | 13 (21.7%) |  |
|  | 3000-3900 | 0 (0.0%) | 2 (3.3%) |  |
|  | 4000 and above | 34 (100.0%) | 2 (3.3%) |  |
| Marital Status of Participant | Single | 5 (14.7%) | 28 (46.7%) | < .001 |
|  | Married | 28 (82.4%) | 22 (36.7%) |  |
|  | Widower | 0 (0.0%) | 4 (6.7%) |  |
|  | Separated | 1 (2.9%) | 5 (8.3%) |  |
|  | Others | 0 (0.0%) | 1 (1.7%) |  |
| Ethnic Background | Akan | 20 (58.8%) | 43 (71.7%) | 0.009 |
|  | Mole-Dagbon | 2 (5.9%) | 0 (0.0%) |  |
|  | Ewe | 4 (11.8%) | 3 (5.0%) |  |
|  | Ga-Dangme | 4 (11.8%) | 0 (0.0%) |  |
|  | Guan | 1 (2.9%) | 0 (0.0%) |  |
|  | Grusi | 0 (0.0%) | 2 (3.3%) |  |
|  | Mande | 0 (0.0%) | 0 (0.0%) |  |
|  | Others | 3 (8.8%) | 12 (20.0%) |  |
| Health Insurance Status | Yes | 32 (94.1%) | 50 (83.3%) | 0.132 |
|  | No | 2 (5.9%) | 10 (16.7%) |  |
| Number of Children | No Children | 7 (20.6%) | 13 (21.7%) | 0.161 |
|  | 2 to 3 Children | 21 (61.8%) | 20 (33.3%) |  |
|  | 4 to 6 Children | 6 (17.6%) | 26 (43.3%) |  |
|  | 7 or More Children | 0 (0.0%) | 1 (1.7%) |  |

Note. N = 94. Percentages are rounded to one decimal place. P-values are based on chi-square tests. Monthly income values in local currency units.

**Supplementary Table 2. Factor Loadings of Dietary Patterns Identified Through Factor Analysis**

| **Food Groups** |  | | |
| --- | --- | --- | --- |
|  | **Indigenous Ghanaian Pattern** | **Westernised Pattern** | **High Protein Pattern** |
| Tubers and Plantain | 0.842 | -0.003 | 0.051 |
| Local Sugared Beverages | 0.762 | 0.159 | 0.115 |
| Cereals and Grains | 0.699 | 0.313 | 0.219 |
| Fruits and Fruit Juice | 0.684 | 0.434 | 0.179 |
| Nuts, Seeds, and Legumes | 0.647 | 0.076 | 0.498 |
| Fats, oils, and fat-based foods | 0.522 | 0.339 | 0.384 |
| Sugared Snacks | 0.284 | 0.735 | -0.032 |
| Milk and Milk Products | 0.131 | 0.679 | 0.321 |
| Energy drinks and soft drinks | 0.069 | 0.653 | -0.101 |
| Tea and Coffee | 0.073 | 0.651 | 0.496 |
| Sweats | 0.326 | 0.604 | -0.088 |
| Fish and Seafood | 0.218 | -0.181 | 0.783 |
| Vegetables | 0.317 | 0.039 | 0.696 |
| Meat, Poultry, and Eggs | -0.066 | 0.526 | 0.616 |
| % Variance Explained | 38.405 | 12.868 | 10.140 |
| Cumulative Variance | 38.405 | 51.273 | 61.413 |
| Note. Factor loadings were deducted using PCA with varimax rotation. % Variance Explained reflects the proportion of dietary intake variability accounted for by each pattern. | | | |

**Supplementary Table 3: Multivariate associations between lifestyle factors and the cardiometabolic markers**

| **Cardiometabolic Markers** | **PA**  **aOR (95% CI) P-value** | **Indigenous**  **aOR (95% CI) P-value** | **Westernized**  **aOR (95% CI) P-value** | **High protein**  **aOR (95% CI) P-value** | **JCQ**  **aOR (95% CI) P-value** | **ERI**  **aOR (95% CI) P-value** |
| --- | --- | --- | --- | --- | --- | --- |
| FBS | 1.000 (0.999-1.001) 0.831 | 2.039 (0.903-4.606) 0.087 | 0.496 (0.166-1.476) 0.207 | 1.384 (0.599-3.194) 0.447 | 0.000 (0.000-0.223) 0.015 | 0.172 (0.010-3.018) 0.229 |
| Blood pressure | 1.000 (0.999-1.001) 0.809 | 1.079 (0.611-1.905) 0.794 | 1.197 (0.586-2.443) 0.622 | 1.869 (1.011-3.455) 0.046 | 0.592 (0.010-36.869) 0.804 | 2.261 (0.360-14.204) 0.384 |
| Abdominal obesity | 1.000 (1.000-1.000) 0.920 | 1.052 (0.599-1.848) 0.861 | 0.357 (0.165-0.771) 0.009 | 1.384 (0.818-2.340) 0.225 | 0.168 (0.006-4.687) 0.294 | 1.614 (0.286-9.113) 0.588 |
| High triglycerides | 0.999 (0.998-1.001) 0.235 | 1.558 (0.757-3.206) 0.229 | 0.685 (0.304-1.547) 0.363 | 0.659 (0.310-1.399) 0.277 | 0.079 (0.001-5.017) 0.231 | 1.457 (0.183-11.612) 0.722 |

Adjusted for sex, age, education and income

|  | Westernised Pattern | Indigenous Ghanaian Pattern | High Protein Pattern |
| --- | --- | --- | --- |
| Tubers and Plantain | 0.842 | -0.003 | 0.051 |
| Local Sugared Beverages | 0.762 | 0.159 | 0.115 |
| Cereals and Grains | 0.699 | 0.313 | 0.219 |
| Fruits and Fruit Juice | 0.684 | 0.434 | 0.179 |
| Nuts, Seeds, and Legumes | 0.647 | 0.076 | 0.498 |
| Fats, oils, and fat-based foods | 0.522 | 0.339 | 0.384 |
| Sugared Snacks | 0.284 | 0.735 | -0.032 |
| Milk and Milk Products | 0.131 | 0.679 | 0.321 |
| Energy drinks and soft drinks | 0.069 | 0.653 | -0.101 |
| Tea and Coffee | 0.073 | 0.651 | 0.496 |
| Sweats | 0.326 | 0.604 | -0.088 |
| Fish and Seafood | 0.218 | -0.181 | 0.783 |
| Vegetables | 0.317 | 0.039 | 0.696 |
| Meat, Poultry, and Eggs | -0.066 | 0.526 | 0.616 |

**Supplementary Figure 1. Radar plot showing the dietary distribution**
